# Identification of immune-related mitochondrial metabolic disorder genes in septic shock using bioinformatics and machine learning

**DOI:** 10.1186/s41065-024-00350-y

**Published:** 2024-11-28

**Authors:** Yu-Hui Cui, Chun-Rong Wu, Li-Ou Huang, Dan Xu, Jian-Guo Tang

**Affiliations:** Department of Trauma-Emergency & Critical Care Medicine Center, Shanghai Fifth People’s Hospital, Fudan University, No.801 Heqing Road, Minhang District, Shanghai, 200240 China

**Keywords:** Mitochondria, Differentially expressed genes, Septic shock, Bioinformatics, Machine learning

## Abstract

**Purpose:**

Mitochondria are involved in septic shock and inflammatory response syndrome, which severely affects the life security of patients. It is necessary to recognize and explore the immune-mitochondrial genes in septic shock.

**Methods:**

The GSE57065 dataset was acquired from the Gene Expression Omnibus (GEO) database and filtered by limma and the weighted correlation network analysis (WGCNA) to identify mitochondrial-related differentially expressed genes (MitoDEGs) in septic shock. The function of MitoDEGs was analyzed using the Gene Ontology (GO) analysis, Kyoto Encyclopedia of Genes and Genomes (KEGG), and Gene Set Enrichment Analysis (GSEA), respectively. The Protein-Protein Interaction (PPI) network composed of MitoDEGs was established using Cytoscape. Support Vector Machine Recursive Feature Elimination (SVM-RFE), Random Forest (RF), and Least Absolute Shrinkage and Selection Operator (LASSO) were used to identify diagnostic MitoDEGs, which were validated using receiver operating characteristic (ROC) analysis and Quantitative Real-time Reverse Transcription Polymerase Chain Reaction (qRT-PCR). Furthermore, the infiltration of immunocytes was analyzed using CIBERSORT, and the correlation between diagnostic MitoDEGs and immunocytes was explored using Spearman.

**Results:**

A total of 44 MitoDEGs were filtered, and functional enrichment analysis showed they were associated with mitochondrial function, and the PPI network had 457 nodes and 547 edges. Four diagnostic genes, MitoDEGs, PGS1, C6orf136, THEM4, and EPHX2, were identified by three machine learning algorithms, and qRT-PCR results obtained similar expression levels as bioinformatics analysis. Furthermore, the diagnostic model constructed by the diagnostic genes had fine diagnostic efficacy. Immunocyte infiltration analysis showed that activated immunocytes were abundant and correlated with hub genes, with neutrophils accounting for the largest proportion in septic shock.

**Conclusions:**

In this study, we recognized four immune-mitochondrial key genes (PGS1, C6orf136, THEM4, and EPHX2) in septic shock and designed a novel gene diagnosis model that provided a new and meaningful way for the diagnosis of septic shock.

**Supplementary Information:**

The online version contains supplementary material available at 10.1186/s41065-024-00350-y.

## Introduction

Sepsis is a systemic life-threatening organ failure arising from the invasion of pathogenic microorganisms such as bacteria, mainly due to the patient’s maladjustment to infection [1]. It is a main public health problem and inflammatory disease that affects numerous people worldwide year after year and has an unacceptable mortality rate of between one-sixth and one-third. Infection in sepsis can activate inflammatory response cells, producing and releasing massive inflammatory mediators, leading to tissue damage. Severe disease can result in septic shock and even multiple organ dysfunction syndrome, which causes massive deaths in intensive care units (ICU) worldwide [2]. Several clinical studies have shown that the early diagnosis, treatment, and nursing care of septic shock patients seriously affect their prognosis; timely diagnosis and effective intervention can significantly reduce mortality [3–5].

Mitochondria are pivotal organelles and play a significant role in inflammatory responses; however, excessive inflammatory responses release more inflammatory mediators, impairing the mitochondrial electron transport chain and resulting in damaged mitochondrial function. Mitochondrial damage results in decreased oxidative phosphorylation, decreased ATP synthesis, increased apoptosis, and altered mitochondrial biogenesis [6]. It can also induce high Ca^2+^ influx through the mitochondrial calcium uniporter (MCU), which can generate excess reactive oxygen species (ROS) and release mitochondrial DNA into the cytoplasm. Thus, TLR9/NLRP3 inflammasome and apoptosis are induced [7]. ATP and mitochondrial dysfunction can lead to decreased PGE2 and promote medullary vasoconstriction [8], which in turn induces the exacerbation of septic acute kidney injury (AKI). Lately, various researchers have pointed out that abnormalities of the immune system and mitochondrial-related dysfunction are tightly correlated to the incidence and progression of sepsis and septic shock; however, the mitochondrial genes related to sepsis and septic shock are not well comprehended, so it is necessary to identify and explore the related genes.

As pivotal biomarkers, the key genes and their differential expression provide new insights for the diagnosis of a variety of diseases. Currently, key genes have been identified in several diseases, such as intervertebral disc degeneration [9], fibromyalgia [10], and rheumatoid arthritis [11]. In the context of septic shock, a multitude of studies have identified key genes that are implicated in its pathogenesis. Fan et al. [12]demonstrated that UPP1, S100A9, KIF1B, S100A12, and SLC26A8 exhibit considerable diagnostic value in pediatric septic shock and are associated with immune cell infiltration. Jiang and colleagues have identified FYN and CD247 as potential novel biomarkers for septic shock [13]. Furthermore, Kong et al. [14] indicated that CD177, CLEC5A, CYSTM1, MCEMP1, MMP8, and RGL4 hold significant value for the early diagnosis of individuals with septic shock. However, there is currently a lack of models available for the prognostic prediction of septic shock. Given the severity of septic shock, it is of great clinical significance to construct gene diagnostic models. Key gene screening cannot be separated from an essential artificial intelligence technology—machine learning. It has been widely applied in abundant medical research areas, such as malaria diagnosis [15], cardiovascular disease prediction [16], and the distinction of phenotypic heterogeneity in heart failure [17], which has made a significant contribution to the informatization development of medicine.

Based on bioinformatics and machine learning techniques, we identified a set of genes known as immuneMitoDEGs in septic shock, conducted assessments and validations to determine the diagnostic value and expression levels of these genes, and further explored their correlation with the immune microenvironment.

## Methods

### Data acquisition and preprocessing

GSE57065 and GSE95233 datasets (Table 1) were obtained from the GEO database (https://www.ncbi.nlm.nih.gov/geo/) using the GEOquery package from R software (version 4.1.1, http://r-project.org/) [18]. The GSE57065 dataset consists of 28 samples obtained from patients experiencing septic shock, collected 30 min after the onset of the event, in addition to 25 samples from healthy controls. The GSE95233 dataset encompasses 51 samples from septic shock patients on their first day of hospitalization, along with 22 healthy control samples. Both datasets were produced utilizing the GPL570 [HG-U133_Plus_2] Affymetrix Human Genome U133 Plus 2.0 Array platform. The mRNA expression matrix was obtained via the annotation and normalization of the dataset. Moreover, a total of 1,136 human mitochondrial relevant genes were acquired from the MitoCarta 3.0 database (https://www.broadinstitute.org/mitocarta/mitocarta30-inventory-mammalian-mitochondrial-proteins-and-pathways) to perform subsequent analysis.

**Table 1:** GEO datasets information

GEO ID	Samples (Healthy control: Septic shock)	Sample Source	Platform	Year	Author	Type	Dataset Usage
GSE57065	25:28	Blood	GPL570	2014	Cazalis MA	mRNA	Training set
GSE95233	22:51	Blood	GPL570	2017	Pachot A	mRNA	Validation set

### Identification of differentially expressed mitochondrial genes in septic shock

In this research, the limma package (version 3.60.4) [19] was employed to recognize differentially expressed genes (DEGs) between the septic shock group and the healthy control group. The selection criteria were defined as |logFC| > 1 and p-value < 0.05. Subsequently, the WGCNA package (version 1.72.5) [20] was used to formulate the weighted co-expression network with the aim of identifying the modular gene set highly associated with septic shock. MitoDEGs were obtained through the intersection among DEGs, the WGCNA module gene set, and the mitochondrial-relevant gene set.

### Function enrichment analysis and protein-protein Interaction network construction

GO analysis and KEGG pathway enrichment analysis derived from the clusterProfiler (version 4.12.2) [21] were performed to analyze the biological function of MitoDEGs with the threshold of *p* < 0.05 in the Benjamin-Hochberg test. With “c2.all.v2023.1.Hs.symbols.gmt” in the Molecular Signatures Database (MSIGDB)(https://www.gsea-msigdb.org/gsea/msigdb) as the reference gene set, the GSEA was performed. The PPI network was established using the information obtained from the STRING database (https://cn.string-db.org/) [22], employing a minimum interaction score threshold of 0.9. The network was subsequently visualized using Cytoscape.

### Identification of diagnostic MitoDEGs using machine learning algorithms

In order to obtain the MitoDEGs with diagnostic value, three common machine learning algorithms were used. SVM-RFE [23] is an original machine learning algorithm that can sort features dependent on recursion in order to avert over-fitting [24]. LASSO regression, as a dimensionality reduction method, has a stronger performance in evaluating high-dimensional data than regression analysis and can improve prediction accuracy using regularization. Random Forest (RF) is a type of classifier based on the decision tree algorithm for solving regression and classification problems. Here, the “e1071” package (version 1.7.14), glmnet package (version 4.1.8) [25], and randomForest package (version 4.7.1.2) [26] were used to establish the SVM-RFE model, LASSO model, and RF model, respectively. The average misjudgment rates of the SVM-RFE model and LASSO model were compared using 10-fold cross-validation. The calculated mean decrease Gini index was used to determine feature importance in RF. Subsequently, the MitoDEGs with diagnostic value were obtained through the intersection among the genes acquired from all three models. Finally, the diagnostic model was established using logistic regression and validated using the ROC curves based on the training set (GSE57065) and the validation set (GSE95233). The area under the curve (AUC) was determined using the pROC package (version 1.18.5) [27].

### Expression levels validation of diagnostic MitoDEGs by qRT-PCR

With the aim of validating the expression levels of diagnostic MitoDEGs in samples obtained from septic shock patients, peripheral blood samples were collected using PAXGene™ tubes (PreAnalytiX, Switzerland) from patients with septic shock and healthy volunteers, with the approval of the Ethics Committee and obtaining informed consent from all participants. Firstly, total RNA was extracted using the PAXgene™ Blood RNA kit (PreAnalytiX, Switzerland), followed by reverse transcription to obtain cDNA using TransScript^®^ II First-Strand cDNA Synthesis SuperMix (TransGen Biotech, China). Subsequently, the qRT-PCR reaction system was constructed using ChamQ Universal SYBR qPCR Master Mix (Vazyme Biotech, China), and the experiments were performed to validate the mRNA expression levels of diagnostic MitoDEGs. The 2^−ΔΔCT^ method was employed to calculate the relative expression levels of each gene, with GAPDH as a reference for normalization. The primer sequences were exhibited in Supplemental Table 1.

### Immune cell infiltration analysis

To procure the matrix of immune cell infiltration, the original code and corresponding immune cell files were downloaded from the CIBERSORT website (https://cibersortx.stanford.edu/)[28]. Boxplot and immune-infiltrating cell stacking histograms were plotted utilizing the ggplot2 (version 3.5.1) and ggpubr packages (version 0.6.0), respectively. The corrplot package (version 0.92) was employed to plot the correlation heatmaps in order to visualize the correlation of immune cell infiltration. The correlation between MitoDEGs and immune cell infiltration was analyzed using Spearman and then we used the pheatmap package (version 1.0.12) to visualize the results at last.

### Statistical analysis

All data processing and statistical analysis were executed employing R software (version 4.1.1), and *P* < 0.05 was considered statistically significant.

## Results

### MitoDEGs in septic shock

**Fig. 1:**
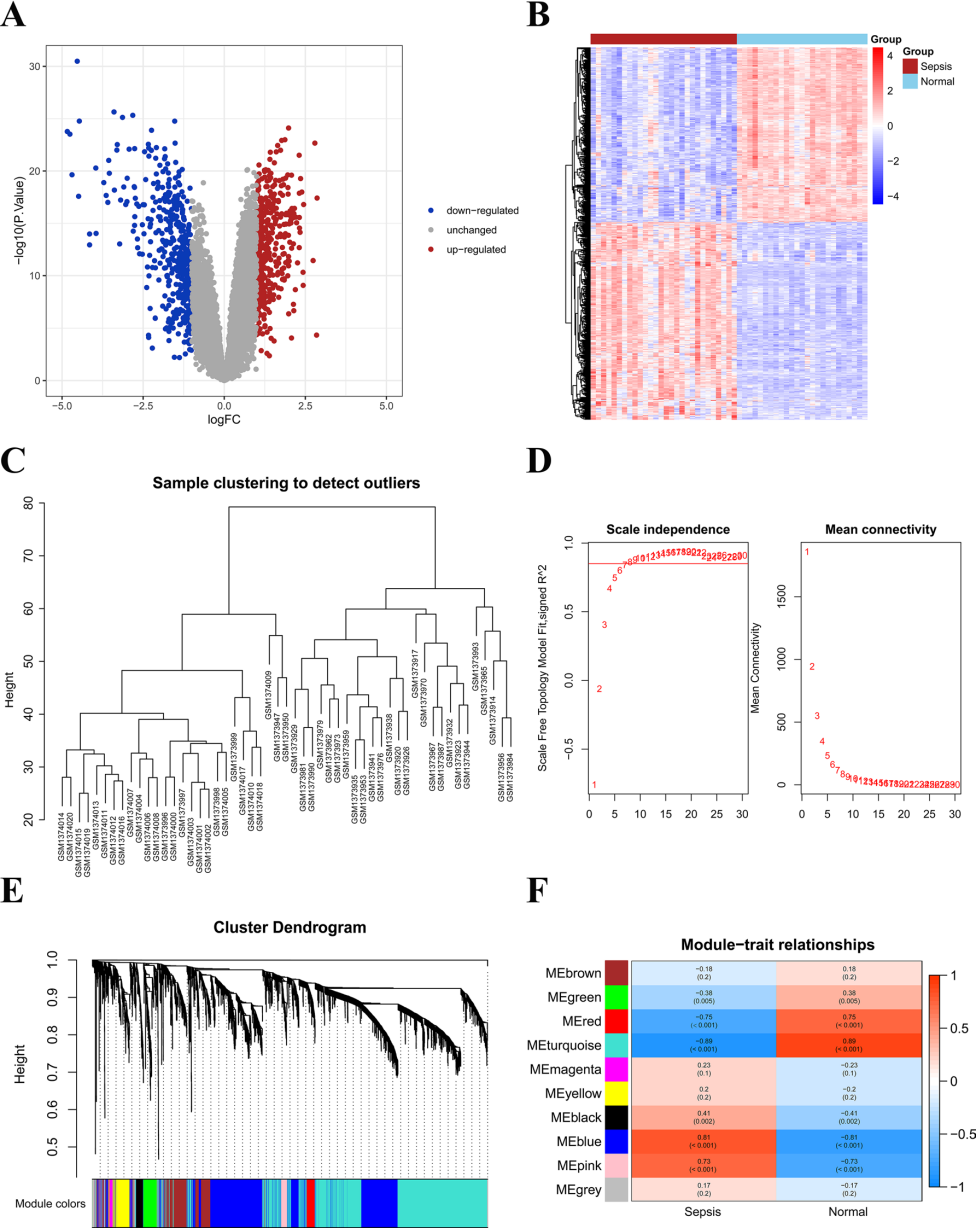
Recognition of DEGs and WGCNA analysis results. (**A**) Differential gene volcano map; (**B**) Differential gene heatmap; (**C**) Sample clustering tree diagram; (**D**) Soft threshold and scale-free topology fitting index; (**E**) Combination module under cluster tree; (**F**) Module trait correlation heatmap. Red represents positive correlations, while blue represents negative correlations

**Fig. 2:**
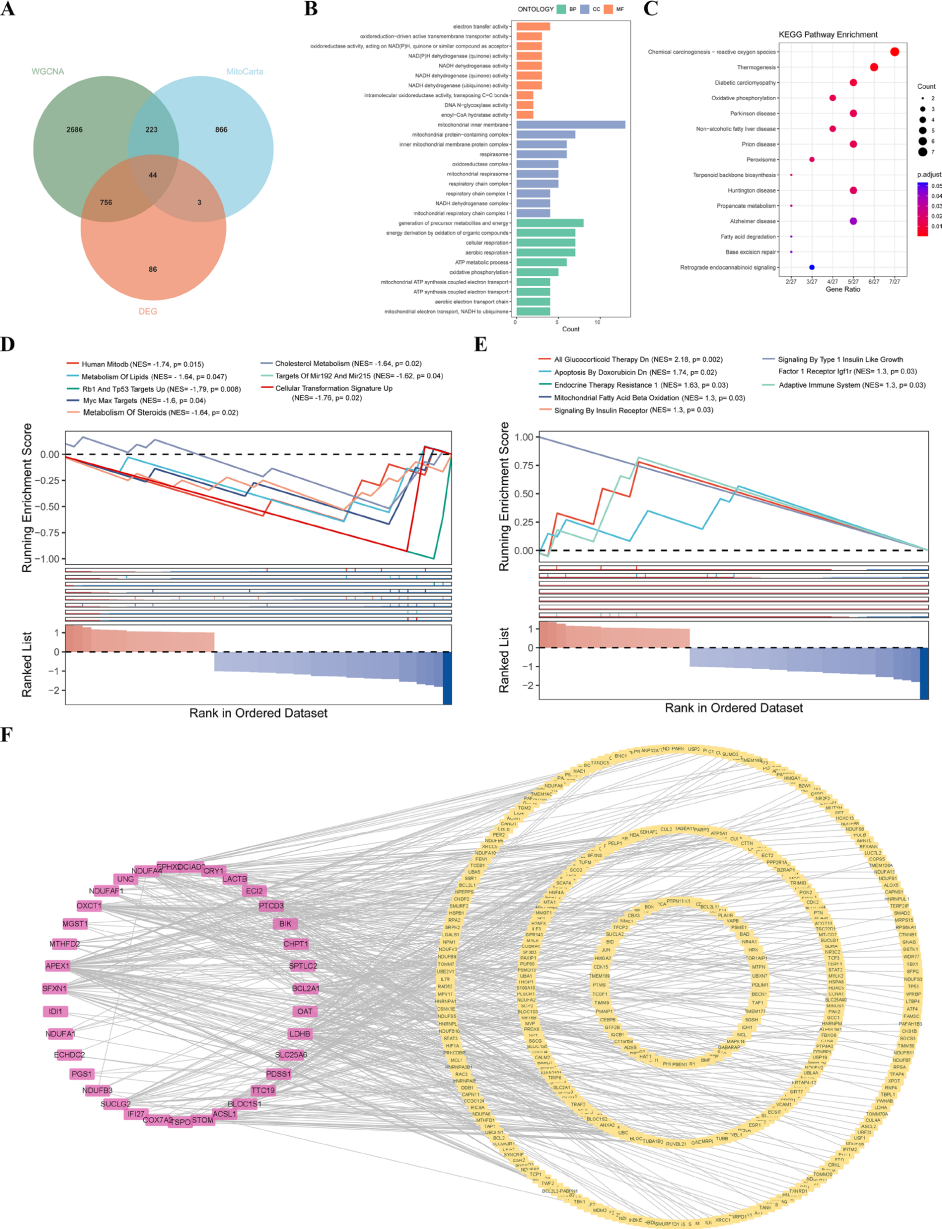
The functional enrichment analysis results and the PPI network composed of MitoDEGs. (**A**) Venn diagram of MitoDEGs; (**B**) GO analysis results; (**C**) KEGG analysis results; (**D**) Down-regulated pathway in septic shock (GSEA); (**E**) Up-regulated pathway in septic shock (GSEA); (**F**) PPI network constructed by MitoDEGs

Compared with the healthy control, we found a total of 889 DEGs in septic shock samples, 417 genes were over-expressed while 472 genes were under-expressed (Fig. 1A - B). No significantly abnormal samples were found after clustering the healthy samples and the septic shock samples (Fig. 1C). As shown in Fig. 1D, when R^2^ > 0.85 and the average connectivity was high, the soft threshold was set to 8. The initialized and merged modules were exhibited under the cluster tree (Fig. 1E), and 10 modules were identified after combining powerfully correlated modules using the 0.25 cluster height limit. As shown in Fig. 1F, the blue module containing 1,540 genes was significantly positively associated with septic shock (*R* = 0.81, *p* < 0.001), and the turquoise module containing 2,169 genes was significantly negatively associated with the disease (*R* =-0.89, *p* < 0.001). Thus, WGCNA analysis identified that both the blue module and the turquoise module were associated with septic shock. Finally, a total of 44 MitoDEGs were obtained through the intersection among DEGs (889), key module genes (3,709), and mitochondria-relevant genes (1,136) (Fig. 2A).

### Enrichment analysis and protein-protein Interaction networks

Afterwards, the functional enrichment analysis of the MitoDEGs was conducted. The results of GO enrichment (Fig. 2B) indicated that the prominent terms were mostly about mitochondria, including aerobic respiration (biological processes, BP), mitochondrial inner membrane (cell components, CC), and electron transport activity (molecular functions, MF), etc. The results of KEGG enrichment mainly involved pathways mostly related to mitochondrial activities as well, for example, Chemical carcinogenesis-reactive oxygen species. Meanwhile, disease-related pathways such as nonalcoholic cirrhosis and diabetic cardiomyopathy were identified (Fig. 2C). The results of GSEA demonstrated that the metabolic pathways and other pathways, including Human_Mitodb, Rb1_and_Tp53_Targets_Up and Cellular_Transformation_Signature_Up were down-regulated (Fig. 2D), while the pathways involved in several diseases were up-regulated in septic shock (Fig. 2E). The PPI network of MitoDEGs (Fig. 2F) had 457 nodes and 547 edges in total.

### Selection of diagnostic MitoDEGs

**Fig. 3:**
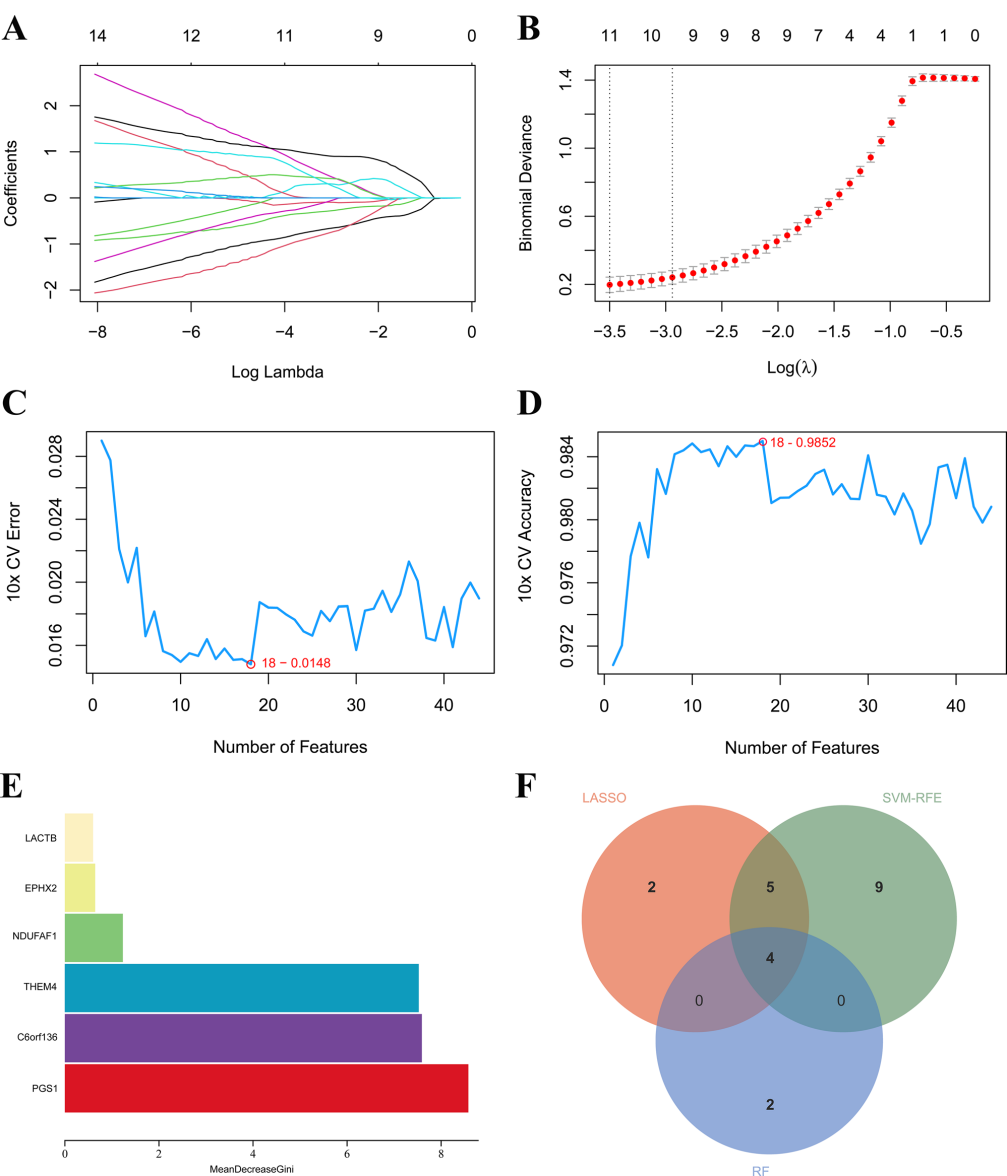
Identification of d-MitoDEGs using three machine learning algorithms. (**A**, **B**) Path maps of regression coefficients and cross-validation curves in the LASSO; (**C**, **D**) Change curves for prediction accuracy and error values for every gene in the SVM-RFE; (**E**) Significance of characteristic genes in the random forest algorithm; (**F**) Wayne map of the characteristic genes acquired by the three algorithms

For the follow-up potential genetic diagnostic studies, we used three frequently used machine learning algorithms (SVM-RFE, RF, and LASSO) to obtain the MitoDEGs with diagnostic value. Through the recursive elimination of insignificant features, SVM-RFE effectively identifies the most representative subset of features, making it particularly advantageous for high-dimensional data. LASSO regression incorporates an L1 regularization term, which facilitates the automatic selection of significant features associated with the target variable, thereby rendering it especially suitable for high-dimensional datasets. The RF model is particularly well-suited for managing high-dimensional data and maintains a considerable degree of accuracy even in the absence of training data, demonstrating robust generalization capabilities. Furthermore, during the training phase, RF demonstrates a high level of proficiency in evaluating the significance of individual features, thereby facilitating the feature selection process. Through the application of the aforementioned three methods for feature selection, in conjunction with an analysis of their intersection, facilitated the effective identification of reliable feature genes that are significantly associated with septic shock. A total of 11 septic shock-related genes were filtered by the LASSO algorithm (Fig. 3A - B), and 18 best-candidate genes for septic shock were identified by SVM-RFE (Fig. 3C - D). We then used a random forest algorithm to determine the importance of features and selected five genes as diagnostic genes (Fig. 3E). Finally, the candidate genes obtained from the three machine learning algorithms were intersected; thus, four diagnostic MitoDEGs (d-MitoDEGs) were identified, namely PGS1, C6orf136, THEM4, and EPHX2 (Fig. 3F).

### Validation of diagnostic MitoDEGs

**Fig. 4:**
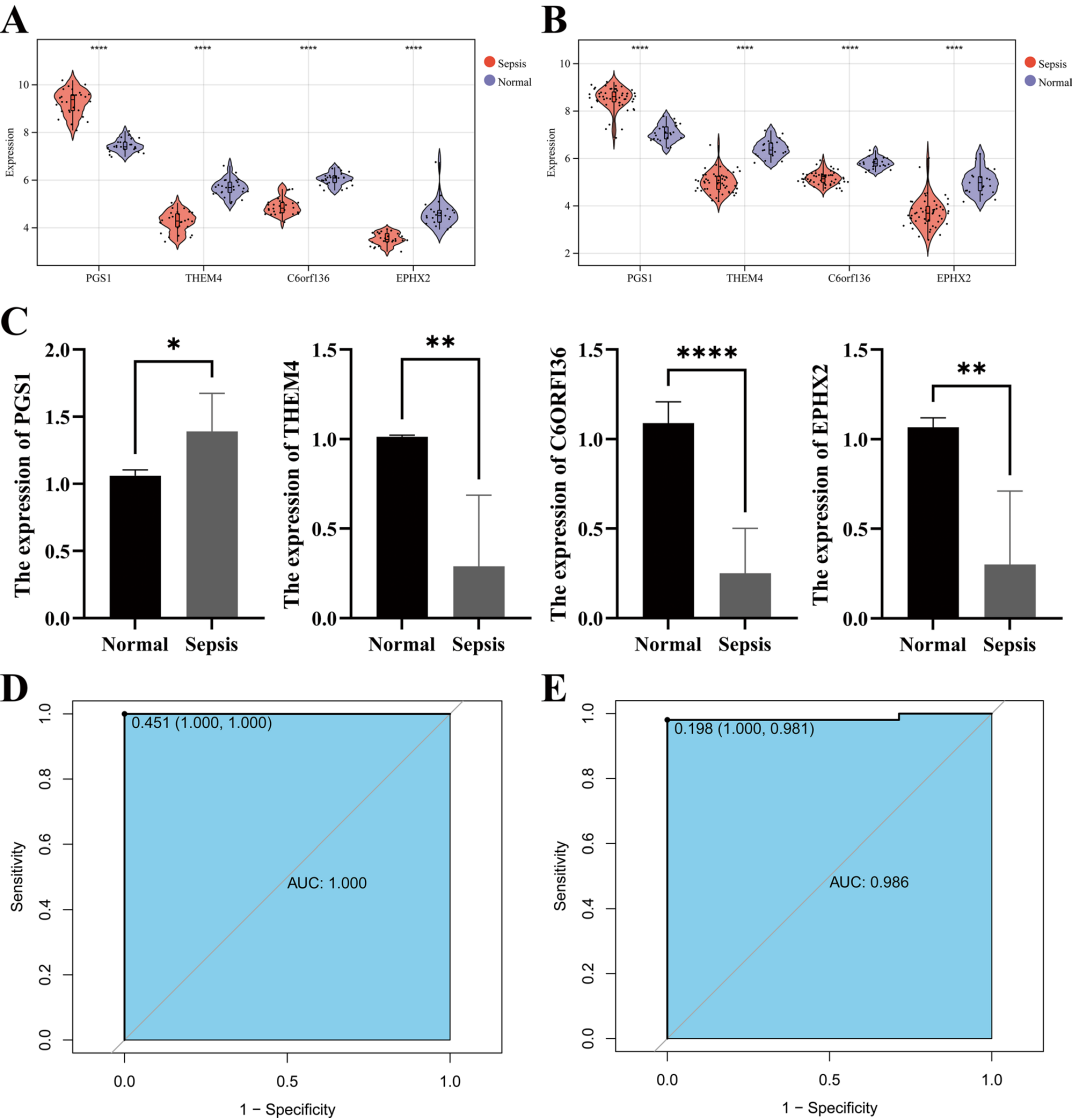
Differential expression of d-MitoDEGs and ROC curves. (**A**) The differences in d-MitoDEGs expression between the SS group and the control group in the training set; (**B**) Validation of the differences of d-MitoDEGs expression between the SS group and the control group; (**C**) Validation of the expression levels of d-MitoDEGs between the SS group and the control group using qRT-PCR experiment, *p < 0.05;**p < 0.01; ****p < 0.0001; (**D**) ROC curve of the diagnostic model in the training set; (**E**) ROC curve of the diagnostic model in the validation set

After obtaining the d-MitoDEGs, ROC analyses were executed to determine and validate the diagnostic potential of PGS1, C6orf136, THEM4, and EPHX2. In the training set, compared with the control group, PGS1 was significantly overexpressed, while C6orf136, THEM4, and EPHX2 were significantly underexpressed in septic shock (Fig. 4A). Similarly, the expression trends of the four genes in the validation set mirrored those observed in the training set (Fig. 4B). Similar expression levels were also validated in the blood samples from patients using qRT-PCR experimental validation (Fig. 4C). Subsequently, the diagnostic model for d-MitoDEGs was constructed using logistic regression. Four genes were involved in the regression model, and the results manifested that the regression coefficients of PGS1, THEM4, and C6orf136 were 0.2308, -0.1515, and -0.2032, respectively, with the P values all less than 0.05. However, the P value of the EPHX2 gene was not significant, so the diagnostic model of three genes was constructed as follows: risk score = (0.2308 * PGS1) + (-0.1515 * THEM4) + (-0.2308 * C6orf136). The ROC curves of the diagnostic model in the training set and the validation set are shown in Fig. 4D - E, and the AUROCs were 1.000 and 0.986, respectively. We can affirm that this model had good diagnostic efficacy in septic shock.

### Immune infiltration in septic shock

**Fig. 5:**
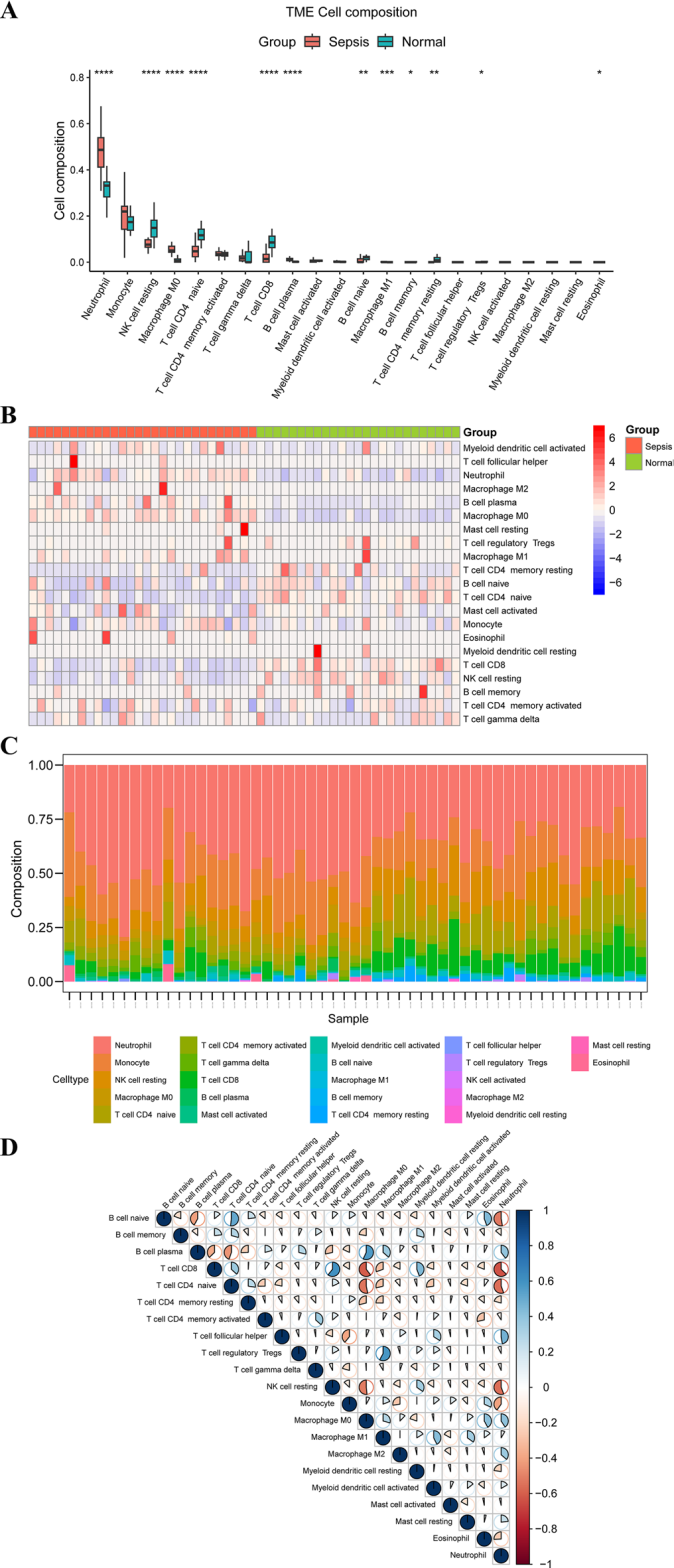
Results of immune cell infiltration analysis; (**A**) Box plot of the proportions of immunocytes between the SS group and the control group, *p < 0.05;**p < 0.01; ***p < 0.001; ****p < 0.0001; (**B**) Heat map of immunocyte ratios between the SS group and the control group; (**C**) Stacked histogram of immunocytes; (**D**) Heat map of the correlation between immunocytes, with blue demonstrating the positive correlation and red demonstrating the negative correlation

To further explore the correlation between immunization activities and septic shock, CIBERSORT algorithms were employed to carry out the immune infiltration analysis, which showed that all 21 immunocyte types were infiltrated, except activated NK cells, with significant differences in 12 immunocyte types between the SS group and the control. Specifically, neutrophils, M0 macrophages, M1 macrophages, plasma B cells, memory B cells, and eosinophils were more abundant in septic shock, whereas quiescent NK cells, naive CD4 ^+^ T cells, CD8 ^+^ T cells, memory quiescent CD4 ^+^ T cells, naive B cells, and Tregs were more abundant in the control group (Fig. 5A - B). The percentage of immunocytes was calculated, resulting in neutrophils being the cell type with the highest percentage, and then the stacked histogram (Fig. 5C) was plotted. The correlation heatmap of 21 immunocyte infiltrations (Fig. 5D) indicated that neutrophils were significantly negatively associated with naive B cells, CD8 ^+^ T cells, naive CD4 ^+^ T cells, resting NK cells, and monocytes, while positively associated with plasma B cells, follicular helper B cells, M0 macrophages, and M2 macrophages.

### Correlation between immune cells and diagnostic MitoDEGs

**Fig. 6:**
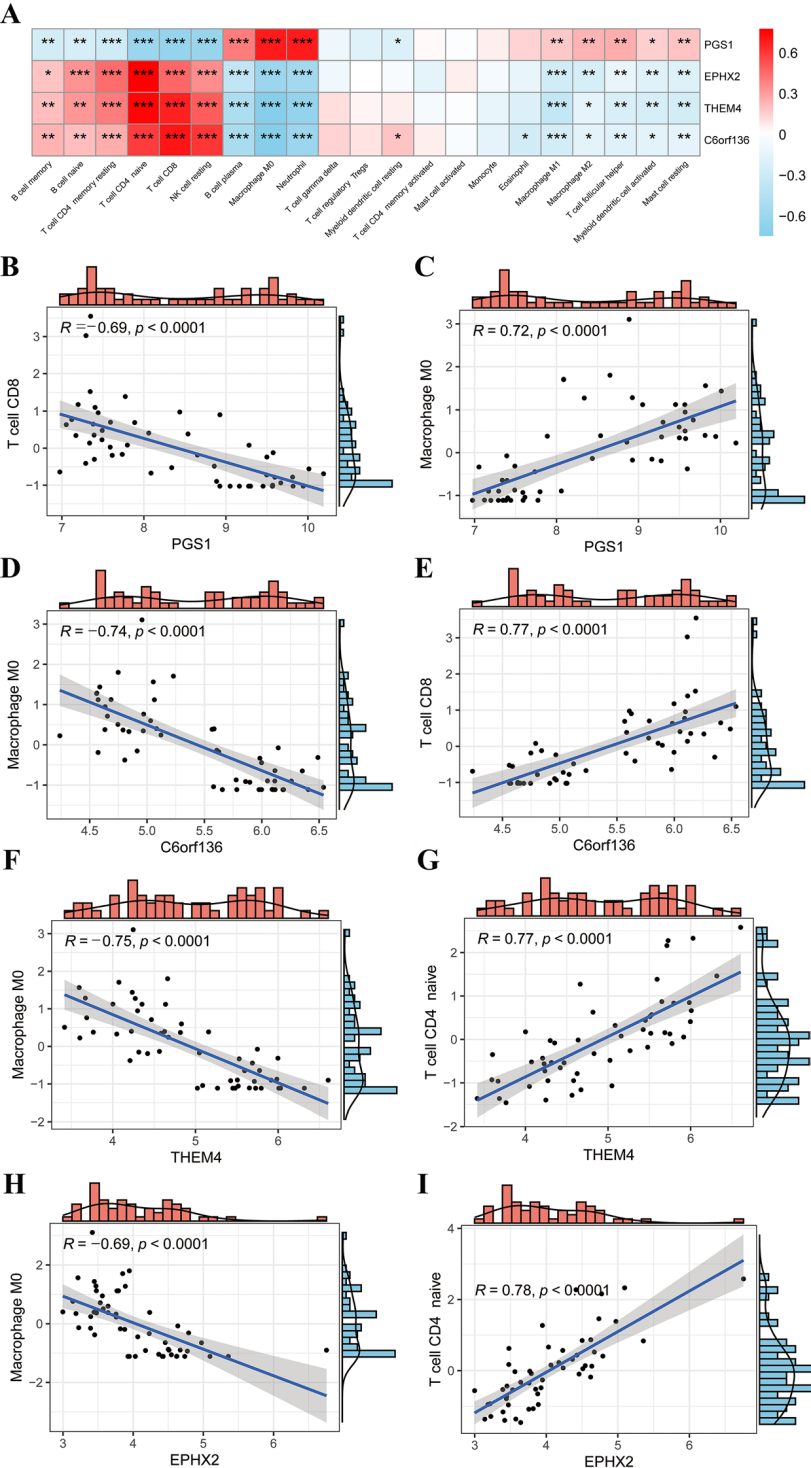
Correlation between immunocytes and d-MitoDEGs. (**A**) Heatmap of the correlation between d-MitoDEGs and immunocytes, with red demonstrating positive correlations and blue demonstrating negative correlations. *P < 0.05; **p < 0.01; ***p < 0.001; ****p < 0.0001; (**B**, **C**) Correlation scatter plots of PGS1 with CD8 ^+^ T cells and M0 macrophages; (**D**, **E**) Correlation scatter plots of C6orf136 with M0 macrophages and CD8 ^+^ T cells; (**F**, **G**) Correlation scatter plots of THEM4 with M0 macrophages and naive CD4 ^+^ T cells; (**H**, **I**) Correlation scatter plots of EPHX2 with M0 macrophages and naive CD4 ^+^ T cells

The results of the Spearman analysis indicated the correlation between immune cells and d-MitoDEGs, as shown in Fig. 6A. Specifically, PGS1 showed a positive correlation with neutrophils and M0 macrophages, while it had a negative correlation with CD8 ^+^ T cells and naive CD4 ^+^ T cells. C6orf136 was positively correlated with CD8 ^+^ T cells and CD4 ^+^ T cells, but it had a negative association with M0 macrophages. THEM4 demonstrated a positive relationship with CD8 ^+^ T cells and naive CD4 ^+^ T cells, but it had a negative correlation with M0 macrophages and neutrophils. EPHX2 was positively associated with naive CD4 ^+^ T cells but negatively associated with M0 macrophages and neutrophil cells. The scatter plots of the correlation between the four d-MitoDEGs and immunocytes with the strongest relevance are shown in Fig. 6B-I.

## Discussion

Sepsis is a catastrophic organ dysfunction caused by the invasion of pathogenic microorganisms into the host body and the host’s imbalanced response to infection, which can deteriorate into septic shock [29]. Nowadays, the mortality rate remains high, although research on sepsis has become increasingly in-depth. One of the main reasons for this is the lack of a detailed understanding of the pathogenesis and pathological process of sepsis at the genetic level [30]. Furthermore, numerous challenges have been encountered in the diagnosis and treatment of sepsis and septic shock. First, sepsis is a syndrome with uncertain pathophysiological characteristics; hence there exist a multitude of widely held complicated problems for its diagnosis. Secondly, the “traditional” diagnostic criteria of sepsis, “2 or more SIRS criteria” [31] are no longer used at present, while the diagnostic method “Sequential [Sepsis-related] Organ Failure Assessment (SOFA) [32] score increasing”, which was recommended by a sepsis expert panel in 2016 [1], is also a bit cumbersome. Therefore, the lack of high-quality diagnostic markers for sepsis has been fully exposed to researchers, making it extremely essential to explore potential biomarkers for septic shock.

Plenty of previous studies have confirmed the inseparable correlation between the pathological process of sepsis and the immune response and mitochondrial behavior [33]. McCall et al. [34] confirmed that sepsis caused phosphorylation and inactivation of the mitochondrial pyruvate dehydrogenase complex, which interfered with the tricarboxylic acid cycle and altered the electron transport chain, ultimately leading to an energy imbalance and immune metabolic “paralysis”. The excessive inflammatory response caused by sepsis also activated the mitochondria to produce numerous reactive oxygen species, leading to vascular oxidative stress, and thus septic shock [35]. However, little is known about immune-mitochondrial related genes and their mechanisms, so it is challenging to intervene in sepsis and septic shock from the perspective of diagnosis and therapy of mitochondrial genes. Hence, it is enormously necessary to comprehensively identify and screen out immune-mitochondrial related genes and to deeply explore their mechanisms and related pathways.

Depending on bioinformatics and machine learning, our study identified and filtered immune-related mitochondrial differentially expressed genes and explored their biological function and diagnostic value. Firstly, we analyzed and screened the GEO dataset to obtain a total of 44 mitochondrial differentially expressed genes (MitoDEGs) in septic shock. The potential biological functions and related pathways of MitoDEGs were further analyzed, and the PPI network was established using the STRING database. The analysis results showed that MitoDEGs were correlated with multiple mitochondrial activities, suggesting that they may have a key effect on the occurrence and exacerbation of septic shock. Then, we filtered four d-MitoDEGs (PGS1, C6orf136, THEM4, and EPHX2) utilizing three machine-learning algorithms. ROC analysis verified that the diagnostic model based on d-MitoDEGs had fine diagnostic performance in septic shock. Finally, we analyzed the immune cell infiltration in septic shock and explored the intricate association between immune infiltration and d-MitoDEGs. The results showed that 21 types of immunocytes were infiltrated, and the infiltration of 12 types of immunocytes was significantly different in septic shock. Four d-MitoDEGs were respectively correlated with part of immune cell infiltration.

Among the four hub genes identified in our study, the PGS1 gene primarily encodes phosphatidylglycerophosphate synthase 1, which is anticipated to enhance the activity of CDP-diacylglycerol-glycerol-3-phosphate 3-phosphatidyltransferase and to interact with calcium ions. Furthermore, it is projected to play a significant role in the biosynthetic process of cardiolipin and the metabolic process of diacylglycerol. Currently, there remains a limited number of reports regarding the role of PGS1 in sepsis and septic-related diseases. Zhang et al. [36]developed co-expression networks involving mRNA-lncRNA and mRNA-lncRNA-pathway interactions that include PGS1 mRNA within the context of pediatric sepsis. Furthermore, Li et al. [37]demonstrated that six genes, including PGS1, are integral to mitochondrial metabolic dysregulation and immune infiltration in septic cardiomyopathy, and developed a diagnostic nomogram model utilizing these genes to facilitate the early diagnosis of this severe condition. To the best of our knowledge, this study presents, for the first time, the potential risk prediction ability of PGS1 in septic shock and suggests that it plays a significant role in associated immune infiltration. The thioesterase superfamily member 4 (THEM4) gene is expressed in many tissues of the human body, but similarly, there is no report to explain the relationship between the THEM4 gene and septic shock. Up to now, there is a little bit of understanding of C6orf136, which has only been reported in head and neck squamous cell carcinoma [38] and pancreatic adenocarcinoma [39]. Only EPHX2 has been identified as a differentially expressed lipid metabolism-related gene responsible for immune dysfunction in sepsis [40] and speculated as a potential biomarker for sepsis-associated acute kidney injury (SA-AKI), exhibiting decreased expression in cases of SA-AKI [41], which aligns with our findings.

Gene diagnostic models are algorithms or machine learning models that analyze genetic data to identify or predict the presence of certain genetic variations or diseases. As an emerging method of predicting diseases, they vigorously promote the development of precision medicine and have significant assistance in guiding clinicians in the rapid diagnosis of diseases. The combination of gene diagnostic models with biomarkers obtained from bioinformatics and machine learning techniques improves the accuracy of disease diagnosis, for example, osteoarthritis (OA). It has been confirmed that early detection, diagnosis, and therapy can effectively improve prognosis, while gene diagnostic models can effectively help with rapid diagnosis and early intervention. Therefore, Han et al. [42] focused on the establishment of a 5-gene diagnostic model using support vector machines and verified that it had good diagnostic performance. In addition, there have been abundant reports of gene diagnostic models for diseases such as thyroid cancer [43]and multiple sclerosis [44]. Nevertheless, as far as we know, no report on the establishment of a gene diagnostic model related to septic shock has been published, so our work is of certain importance and inspiration for the clinical diagnosis of septic shock.

As we all know, sepsis and septic shock are closely associated with dysregulations in the immune system. Therefore, this study undertook a comprehensive analysis of immune cell infiltration within the context of septic shock. The analysis revealed a significant increase in the infiltration of neutrophils, M0 macrophages, M1 macrophages, plasma B cells, memory B cells, and eosinophils in septic shock. Several decades ago, researchers identified that the pathogenesis of septic shock is closely associated with the activation of neutrophils [45]. A significant influx of neutrophils rapidly migrates across the endothelium to reach the site of infection, where they exhibit a delay in apoptosis [46], thereby inducing extensive neutrophil infiltration. Furthermore, the significant influx of pathogenic bacteria into the circulatory system of patients experiencing sepsis and septic shock leads to a substantial accumulation and infiltration of M0 macrophages, which possess phagocytic capabilities, as well as M1 macrophages that facilitate inflammatory responses [47]. This observation also aligns closely with the findings of our analysis. This study not only delineated a comprehensive profile of immune cell infiltration in septic shock through bioinformatics analysis but also examined and elucidated the relationship between the identified hub genes and immune cells, thereby providing valuable insights into the mechanisms underlying immune system dysfunction associated with septic shock. In summary, combined with the expression characteristics of d-MitoDEGs and the ROC validation results obtained in our study, we concluded that PGS1, C6orf136, THEM4, and EPHX2 were key genes associated with immune-mitochondria in septic shock. The diagnostic model constructed according to hub genes may provide novel insights and methods for a more accurate clinical diagnosis of septic shock.

## Electronic supplementary material

Below is the link to the electronic supplementary material.

Supplementary Material 1

## Data Availability

No datasets were generated or analysed during the current study.
