# Supplementary material for: Identification of immune-related mitochondrial metabolic disorder genes in septic shock using bioinformatics and machine learning
Source: Hereditas. 2024 Nov 28;161:49. doi: 10.1186/s41065-024-00350-y (PMC11603897; doi:10.1186/s41065-024-00350-y)
Supplement: Supplementary file 1 — Supplementary Material 1 [file 41065_2024_350_MOESM1_ESM.docx]

**Supplemental Table 1** Primers sequences for qRT-PCR.

| GENE | | Forward primer | Reverse primer |
| --- | --- | --- | --- |
| PGS1 | GAGCGCTTCAACGAGACCAT | ACGCTGTTGTCGAAGAGGTACA |  |
| THEM4 | ATTCATGGAGGTGCCATTGC | CCAGCCATCATTGCACACAT |  |
| C6orf136 | CGAGCATTACCGGACCTATGAT | CAAATGAGGCCACTGGAATTC |  |
| EPHX2 | TAAACTGGGCCTCTCTCAAGCA | AGCCATGTACCACACCAGCAT |  |
| GAPDH | GAAGGTGAAGGTCGGAGTC | GAAGATGGTGATGGGATTTC |  |
